# Highly luminescent water-dispersed silicon quantum dots for fluorometric determination of oxytetracycline in milk samples

**DOI:** 10.3906/kim-2007-4

**Published:** 2020-12-16

**Authors:** Hayriye Eda ŞATANA KARA, Burak DEMİRHAN, Buket ER DEMİRHAN

**Affiliations:** 1 Department of Analytical Chemistry, Faculty of Pharmacy, Gazi University, Ankara Turkey; 2 Department of Pharmaceutical Basic Sciences, Faculty of Pharmacy, Gazi University, Ankara Turkey

**Keywords:** Silicon quantum dots, oxytetracycline, fluorescent probe, inner filter effect

## Abstract

A fluorescent probe based on silicon quantum dots (SiQDs) was developed for the selective and sensitive detection of oxytetracycline (OTC) via the inner filter effect (IFE). The water-soluble fluorescent SiQD was synthesized based on the reaction of 3-Aminopropyltriethoxysilane (APTES) and sodium citrate as precursors by the one-pot hydrothermal process. The strong fluorescence emission of quantum dots (QDs) was obtained at 440 nm when excited at 350 nm and OTC had a broad absorption band between 200 and 400 nm. The excitation spectrum of SiQDs was completely overlapped with the absorption spectrum of OTC. The light at an excitation wavelength of QDs absorbed by OTC caused a decrease in fluorescence intensity with an increase in the concentration of OTC. Under optimal conditions, the linear concentration range was 0.92–9.2 µg mL1 with a detection limit (LOD; S/N = 3) of 0.19 µg mL ^-1^ . The proposed method was applied to the determination of OTC in milk samples and satisfactory recoveries (98.8–100.5%) with low RSD % values (0.93–2.31%) were achieved. This simple, selective, sensitive, rapid, and cheap method can be used as a promising tool for OTC analysis in food safety.

## 1. Introduction

Oxytetracycline (OTC), 4-(dimethylamino)-1,4,4a,5,5a,6,11,12a-octahydro-3,5,6,10,12,12a-hexahydro-6-methyL ^-1^ ,11-dioxo-2-naphthacenecarboxamide, belongs to tetracycline-class antibiotics derived from *Streptomyces* spp. [1,2]. This antibiotic has a broad spectrum with a bacteriostatic effect against most gram-negative and gram-positive bacteria. OTC is widely used in humans, animals, vegetables, and fruits to prevent and treat bacterial diseases. However, incorrect and uncontrolled use of OTC can cause allergies, drug resistance, and toxic effects in humans [3]. In addition, poor absorption of OTC from the gastrointestinal system causes a high concentration of drug in the intestine, which leads to change in metabolic activity and microflora properties 1Fernandez H, Miller M (1998). Tetracyclines: Oxytetracycline, chlortetracycline, and tetracycline (addendum). World Health Organization, Geneva 1998 [online]. Website http://www.inchem.org/documents/jecfa/jecmono/v041je07.htm [accessed 19 November 2019].
. One of the areas of the common use of OTC is the treatment or prevention of mastitis and metritis in cows [4]. Most of the tetracyclines applied in cows are excreted with milk. Therefore, OTC residues may be present in milk [5]. World Health Organization (WHO) and Food and Drug Administration (FDA) established a maximum residue limit of 0.1 mg L ^-1^ and 0.3 mg L ^-1^ , respectively, for OTC in milk to protect human health 2Anonymous (2000). FAO/WHO Codex Alimentarius Commission. Risk analysis principles and methodologies of the codex committee on residues of veterinary drugs in foods. 28-31 Mar 2000, Sess. 12 Washington, DC (USA) [online]. Website http://www.fao.org/tempref/codex/Meetings/CCRVDF/ccrvdf12/rv00_06e.pdf [accessed 19 November 2019].
.

In the literature, there are many different analytical methods for the detection of OTC such as electrochemical [3], high performance liquid chromatography (HPLC) with MS, UV, and chemiluminescence detector [6–8], capillary electrophoresis [9,10], spectroscopy [11–13], immunoassays [14], enzyme-link immunosorbent assay (ELISA) [15]. Although the methods mentioned above have high sensitivity and selectivity, they have complicated operation procedures and are not simple and fast enough for rapid detection. Also, they need sample preparation steps such as solid phase extraction, solid liquid extraction, evaporation, and heating [14,16]. In addition, spectrophotometric methods for the determination of OTC in pharmaceuticals or biological samples are based on derivatization of drug with oxidizing or chelating agent. All these tedious clean-up procedures and derivatization steps can bring about errors, waste of time, and extra cost. For this reason, it is needed to develop accurate, simple, and time- and cost-effective analytical methods to detect OTC residues below the maximum permissible residue limits.

In recent years, fluorescence and room temperature phosphorescence (RTP) methods based on the measurement of quantum dots (QDs) signal have gained a great interest. Quantum dots are semiconductor nanoparticles and possess remarkable luminescence emission properties due to their quantum confinement effect. The excitation spectrum of QDs is typically wide and continuous, while the emission band is narrow, symmetrical, and size-tunable. They have also negligible photobleaching and high photochemical stability. Due to these advantages, QDs are widely used as sensors for the detection of ions or neutral molecules in the environment, food, and biological samples [17,18].

In recent years, silicon quantum dots (SiQDs), as new types of photoluminescent nanoparticles, have attracted interest in the field of research due to their properties such as good solubility in water, low toxicity, stable photoluminescence, good biocompatibility, and wide absorption spectrum. Because of these advantages, this type quantum dots have been widely used in fluorescence imaging and detection [19,20]. Literature research has shown that Si quantum dots are generally prepared with electrochemical [21], hydrothermal [22], ultraviolet irradiation [23], and microwave [24] methods. The quantum dots synthesized with these methods are used either as is or modified.

In this research, we developed a simple and rapid SiQDs probe based on the inner filter effect (IFE) to detect OTC residues in milk samples. One of the quenching mechanisms, IFE was discovered by Stokes [25]. Overlapping of the absorption spectrum of the quencher and the excitation or emission spectra of the donor causes the IFE phenomenon. This type of nonirradiation energy conversion can be classified in two ways; i) primary and ii) secondary. In the primary inner filter effect (pIFE), absorbers absorb the light at the excitation wavelength of the fluorophore whereas the secondary inner filter effect (sIFE) refers to the absorption of emission light of fluorophore [26].

Here, eco-friendly SiQDs were prepared using 3-Aminopropyltriethoxysilane (APTES) as the silicon source and sodium citrate as a reducing agent through the hydrothermal process. The synthesized SiQDs have high luminescent and water-solubility character. Moreover, unlabeled QDs allow the determination of OTC from samples with high sensitivity and selectivity. When compared with similar previous studies, the proposed method has a wider linear range and does not need to take a ratio of different emission wavelengths [27,28]. The overlap between the absorption spectrum of OTC and the excitation spectrum of SiQDs allowed IFE-based quantification of OTC. The proposed fluorescent method was successfully applied to detect OTC in milk samples with satisfactory results.

## 2. Materials and methods

### 2.1. Reagents and solutions

3-Aminopropyltriethoxysilane (APTES, 99%), sodium citrate tribasic dihydrate (≥99.0%) and sodium hydroxide were purchased from Sigma–Aldrich (USA), and phosphoric acid and trichloroacetic acid were bought from Merck (Darmstadt, Germany). Oxytetracycline was kindly supplied by I.E. Ulagay Pharm. Ind. (İstanbul, Turkey).

Deionized water was used for the preparation of phosphate buffer (0.02 M). The pH of the buffer solution (pH 7.4) was adjusted using sodium hydroxide (5 M). Aqueous solutions were prepared by deionized water (18.2 MΩ. cm, Simplicity, Milli–Q Millipore water purification system). All the reagents were of the analytical grade and used without further purification.

In this study, commercial cow milk samples were collected from local markets in Ankara, Turkey and were stored in a refrigerator until analysis.

### 2.2. Characterization

The fluorescence excitation and emission spectra were recorded using a Varian Cary Eclipse spectrofluorimeter with a 10 × 10 mm quartz cuvette. Excitation wavelength and excitation/emission bandwidths were 350 nm and 5/10 nm, respectively. PMT voltage was 600 V. Xenon flash lamp was chosen as the light source. UV–Vis absorption spectra were obtained with a Specord 50 Plus (Analytik Jena, Germany) with a 10 × 10 mm path length quartz cell. Absorbance spectra were recorded at 200–900 nm at a speed of 50 nm/s. Particle size was measured using a Malvern Nano Zetasizer ZS90 (Malvern, United Kingdom). The pH values were measured using a combined pH electrode with an Orion model 720 A pH meter. The relative PLQYs of the prepared SiQDs were measured with quinine sulfate in 0.1 M H _2_ SO _4_ as a reference standard. All experiments were performed at room temperature.

### 2.3. Synthesis of SiQDs

The water-soluble fluorescent SiQDs were synthesized by a hydrothermal process with using 3-Aminopropyltriethoxysilane (APTES) and sodium citrate as precursors as per a published method with minor modification [27]. In a typical experiment, 1.2 g of sodium citrate was dissolved in 25 mL nitrogen-saturated deionized water. Afterward, 6 mL of APTES was added and stirred homogeneously for 10 min. The resultant precursor solution was transferred into a stainless steel autoclave and heated at 180 °C for 20 h. After cooling to room temperature, the resultant transparent mixture solution was dialyzed against ultrapure water for 24 h to exclude impurities such as APTES molecules and sodium citrate in the solution. Finally, the synthesized and purified SiQDs solution was stored at 4 °C for further use.

### 2.4. Detection of OTC in aqueous solution with fluorometric titration

The stock solution of OTC was prepared as follows: 0.0125 g of OTC was dissolved in 50.0 mL of deionized water. The stock solution was diluted to prepare various concentrations of OTC in buffer solution. The fluorescence intensities of QDs at 440 nm were measured at excitation/emission slits of 5/10 nm, and excitation wavelength of 350 nm in the absence and presence of OTC. For the detection of OTC, an aqueous solution of SiQDs with a volume of 20 µL was mixed with phosphate buffer solution (0.02 M, pH 7.4) in the 1.0 cm quartz cuvette. The mixture was titrated manually by successive additions of the OTC working solution to detect the fluorescence-quenching effect. The aliquot of each addition of OTC solution was 10 mL to avoid a change in the volume. After each addition, the mixture was shaken well and the fluorescence spectra were obtained after an equilibrium time. Fluorometric measurements were done 1 min after the reactions. All experimental measurements were done at room temperature.

### 2.5. Detection of OTC in milk samples

The milk samples were obtained from the local market and pretreated based on the following procedure. Firstly, in order to remove the proteins, 7.5 mL of 20% trichloroacetic acid (TCA) was added to 5.0 mL milk samples and vortexed for 30 s. (Firlabo, SA, Lyon, France). Afterward, the supernatant was collected to remove lipids and filtered through a 0.20 µm membrane filter (Graphic Controls, Germany). The concentration of OTC in milk samples was analyzed by the standard addition method. The milk samples containing different concentrations of OTC were prepared by adding of stock solution with different volumes. OTC determination was applied five times for each sample and the RSD values were calculated.

## 3. Results and discussion

### 3.1. Characterization of the SiQDs

In the present study, we describe a one-pot, green, and cost-effective SiQDs using 3-Aminopropyltriethoxysilane (APTES) as the silicon source and a sodium citrate as precursor. In this process, siloxane molecules were reduced by trisodium citrate to form silicon crystal nuclei during the heating at high temperatures. In previous studies, different silicon sources such as bulk silicon and SiO _2_ were used for the synthesis of QDs [29]. These hydrophobic QDs need functionalization with hydrophilic species such as hydrophilic molecules and polymers. The surface coating of SiQDs increases not only solubility in aqueous solution but also photostability. Here we chose APTES as a water-soluble silicon source which easily reacted with trisodium citrate for the oxidoreduction process.

The SiQDs were characterized by ultraviolet absorption spectrum, fluorescence spectra, and zeta potential analyzer. A previous study showed that prepared QDs were characterized by having a spherical shape and almost uniform in size [27]. As can be seen in Figure 1A, the SiQDs showed a broad UV absorption band with two absorption peaks at 280 and 350 corresponding to the π – π* and n – π* transitions of the SiQDs [20]. Excitation and emission spectra of the synthesized SiQDs indicated that the QDs possess high photoluminescence properties. The emission maximum of the prepared SiQDs was at 440 nm upon excitation at 350 nm (Figure 1B). There was no shift in the emission peak with a change in excitation wavelength. This may be due to the homogeneous surface condition occurring at high temperature in the hydrothermal process and the single transition probability of electron making the emission independent of the excitation wavelength [30]. In addition, both the excitation and emission curves appeared as symmetrical and well-resolved peaks suggesting that the SiQDs were uniform in size. The data obtained are compatible with previous studies [27,31]. The hydrodynamic diameter of the SiQDs obtained using a zeta potential analyzer study confirmed the formation of small-sized QDs with a diameter of 3.6 nm. In addition, the diameter of QDs was calculated using the Brus equation (1).

(1)ΔE(r)=Eg+h28r2(1me*+1mh*)

where ΔE is the emission energy, E _g_ is band gap energy, r is the radius, h is Planck constant, m _e_^*^ is the effective mass of the excited electron, and m _h_^*^ is the effective mass of excited hole [32,33]. The diameter of the prepared SiQDs was calculated at around 2.7 nm. The difference between the diameters obtained from the zeta potential measurement and the equation is due to different surface states of the QD under the study conditions. Briefly, the hydrated SiQDs samples are directly measured by zeta potential analyzer while the theoretical values are used for equation, which causes larger hydrodynamic diameter than that obtained by the equation. Previous studies show that quantum dots with similar particle sizes with similar synthesis pathways were obtained [22,27,31]. The photoluminescence quantum yield of the SiQDs was calculated to be 23.0% using quinine sulphate as a reference [34]. The prepared QDs are very stable in water for at least 6 months without remarkable precipitation in dark at 4 °C.

### 3.2. Optimization of determination conditions

To achieve the highest sensitivity of SiQDs for OTC, determination conditions such as the buffer solution pH and the response time were analyzed. The fluorescence intensity of the prepared QD was investigated at pH 4.0–12.0. It is seen that the emission intensity reached a maximum value at pH 7.4 and was stable in the range of pH 7.4–9.0 (Figure 2A). Further increase in pH caused the quenching of emission. This pH-dependent luminescence character can be attributed to the surface-covered amino groups. Thus, the pH value was chosen as 7.4 in the latter experiment. In addition, the reaction time was optimized. The fluorescence intensity of SiQDs solution was quenched quickly upon the addition of OTC. The response was too rapid and the reaction reached equilibrium within 1 min (Figure 2B). Therefore, 1 min was chosen as the reaction time to the rapid and sensitive determination of OTC throughout the study.

### 3.3. Detection of OTC in solution

The effect of OTC concentration on the fluorescence emission intensity of SiQDs was investigated to determine OTC in milk samples. As demonstrated in Figure 3, the presence of OTC causes luminescence intensity to decrease because of the quenching effect of the drug on QDs fluorescence signal. The inset graphic in Figure 3 presents the linear response between the ratios of fluorescence intensities and the concentration of OTC. A good linear correlation (r2 = 0.999) was observed from 0.92 to 9.2 µg mL ^-1^ drug concentration. F0 and F are the luminescence intensity of SiQDs at 440 nm in the absence and presence of OTC. Parallel experiments were carried out 5 times. The analytical data for the calibration graph are listed in Table 1. In ICH guidelines, different calculation approaches are described to determine the limit of detection (LOD) and limit of quantification (LOQ) 3ICH (2005). Harmonized tripartite guideline: Validation of analytical procedure text and methodology Q2 (R1). International council on harmonization of technical requirements for registration of pharmaceuticals for human use [online]. Website http://academy.gmp-compliance.org/guidemgr/files/Q2(R1).PDF [accessed 19 November 2019].
. The LOD and LOQ values for OTC were calculated as 0.19 and 0.57 µg mL ^-1^ , respectively, which were obtained based on LOD = 3 s/m and LOQ = 10s/m where s is the standard deviation for five replicates and m is the slope of the calibration curve. Food and Drug Administration (FDA) 4FDA (2019). Code of Federal Regulations. 21(6). Revised as of April 1, 2019 [online]. Website https://www.accessdata.fda.gov/ scripts/cdrh/cfdocs/cfcfr/CFRSearch.cfm [accessed 19 November 2019].
allows a maximum OTC concentration of 0.3 μg mL ^-1^ for milk. The LOD value found shows that the proposed method has enough Sensitivity for the detection of OTC in milk samples for the FDA limit.

**Table 1:** Statistical evaluation of calibration data and recoveries of OTC from spiked milk samples detected by the SiQD as a fluorescent probe.

Linearity range (μg mL ^-1^ )			0.92–9.2
Slope			0.08
Intercept			0.92
Correlation coefficient			0.999
SE of slope			2.1 × 10 ^-4^
SE of intercept			0.01
LOD (μg mL ^-1^ )			0.19
LOQ (μg mL ^-1^ )			0.57
Inter-day precision* (RSD%)			0.57
Intra-day precision* (RSD%)			0.45
Spiked amount (μg mL ^-1^ )	Found amount (μg mL ^-1^ )	Recovery (%)*	RSD (%)
1.98	1.99	100.5	0.93
3.97	3.98	100.2	1.23
5.95	5.97	100.3	2.31
7.94	7.84	98.8	1.98

*Mean of the five experiments SE is the standard error, RSD is the relative standard deviation

In order to evaluate the repeatability of the proposed method, fluorescence intensities of five replicates were measured in the same (intraday precision) and following three days (interday precision). The values of inter- and intraday precision were found 0.57 and 0.45, respectively.

Robustness of the developed method was tested by analyzing the emission intensity of SiQDs and SiQDs–OTC solutions under the deliberate changes in the analytical methodology. The deliberate changes in the pH (±0.1) and reaction time (±10 s) were evaluated with recovery values. For the pH experiment, the buffer solution pH was adjusted to 7.30, 7.40, and 7.50. In these solutions, recovery values were 98.9%, 99.8%, and 100.1%. Reaction time was also tested for 50 s, 60 s, and 70 s and recovery values were 99.7%, 99.9%, and 100.1%, respectively. As a result of the robustness study, small changes in the methodology did not affect the optimized luminescence system significantly.

Selectivity, an important performance parameter, is the essence of sensors. The aim of this study was to apply the developed probe to determine OTC in milk samples containing some ingredients which may influence the fluorescence signal. For this purpose, the influence of the kind of antibiotics, ions, amino acids, and proteins was investigated. Here, the fluorescence intensity of SiQDs was quenched in the presence of 2.76 µg mL ^-1^ OTC. As shown in Figure 4, no obvious changes could be observed after the addition of antiviral and antibiotics such as acyclovir, ampicillin, and trimethoprim, amino acids, and proteins such as L-cysteine, dopamine, L-cystine, and creatinine, common ions such as Na+, K+, Ca2+, Cl–, and CO32-, which were 50 times higher than OTC. Therefore, the detection of OTC by the prepared SiQDs as fluorescent probe has shown an excellent selectivity and sensitivity.

### 3.4. Response mechanism

In this study, the quenching effect of OTC on the emission signal of SiQDs was used for the determination of drugs in milk samples. Generally, well-known quenching mechanisms are static and dynamic (collisional) quenching, fluorescence resonance energy transfer, and inner filter effect. In static quenching, fluorophore and quencher molecules form a nonfluorescent molecule at the ground state, while in the dynamic quenching the quencher interacts with the fluorophore at the excited state. Fluorescence resonance energy transfer is describing energy transfer between two light-sensitive molecules, namely donor and acceptor. For the IFE mechanism, the absorption spectrum of the quencher and the excitation or emission spectra of the donor must be overlapped.

According to Beer–Lambert Law, the molar absorption coefficient of OTC was calculated as 4.6 × 104 at 350 nm wavelength, which demonstrated that it was applicable to sensitive determination by IFE.

As shown in Figure 1B, the great overlapping was observed between the excitation spectra of SiQDs and the absorption spectra of OTC. UV–Vis absorption spectra of OTC in the absence and presence of SiQDs and absorption spectra of SiQDs (Figure 1A) were also recorded. No observable variation in the absorption band of OTC and SiQDs was noticed, which further indicated that no complex has been formed between OTC and SiQDs. In addition, it was noticed that there was an overlap between the emission band of SiQDs and the absorption peak of OTC, which means that the emission of QDs could be partially reabsorbed by OTC. However, the shared area was only a small part of the emission region. Besides, when a different excitation wavelength as 425 nm was chosen, where absorption of OTC was negligible, there was no quenching observed. This observation indicated that possible quenching mechanism may be IFE instead of resonance energy transfer.

The fluorescence emission signal of QDs was quenched by adding OTC (Figure 3). As a result, the fluorescence quenching of OTC on SiQDs was caused by the inner filter effect. Therefore, the fluorescence emission intensity decreased with the increasing of the concentration of OTC (Figure 3), providing the detection of OTC was realized.

Quenching mechanisms also can take place by static or dynamic quenching effects. These mechanisms can be investigated using the Stern–Volmer equation (2):

(2)F0F=1+KSV[Q]

where F0 and F are the fluorescence intensities of fluorophore in the absence and the presence of quencher, respectively. KSV is the Stern–Volmer quenching constant and [Q] is the concentration of the quencher. As can be seen in the inset of Figure 3, a good linear relationship between the concentration of OTC and fluorescence ratios was obtained, and KSV value was calculated as 3.9 × 104 M–1 which indicated moderate interaction between SiQDs and OTC. This result explained that the quenching mechanism might be partially due to static quenching. All the obtained results confirmed that the possible quenching mechanism is based on mainly IFE and partially static quenching effect.

### 3.5. Detection of OTC in milk samples

The applicability of the proposed SiQDs fluorescent probe based on IFE was further investigated by measuring spiked milk samples. For this purpose, OTC was spiked to the milk samples at increasing concentrations and treated for measurement. As shown in Table 1, the recoveries of OTC from spiked milk samples ranged from 98.8% to 100.5% and the RSD% values were between 0.93% and 2.31%. Therefore, the obtained results indicated that this Si quantum dots fluorescent probe could be an effective way for rapid and accurate detection of OTC in milk.

There have been a few reports on OTC detection in different samples with using different methods based on chromatography, electrochemistry, capillary electrophoresis, and spectroscopy (Table 2) [10,13,35–40]. Almost all of them need high quantity and quality chemicals, time, cost, and qualified operators. On the contrary, the proposed method is suitable for green chemistry, and compared with other methods, has a wide linear range, enough sensitivity, and high recovery values.

**Table 2:** OTC levels using different methods in different samples.

Samples	Method	Linearity range	LOD	Recovery (%)	References
Capsules	Capillary electrophoresis	80-120%	0.024 mg mL ^-1^	99.3	[10]
Cow’s milk	Midinfrared spectroscopy	10 ^-4^ 00 μg L ^-1^	> 10 μg L ^-1^	99	[13]
Urine	Differential pulse polarography	6.5 x 10 ^-6^ -9.8 x 10 ^-5^ mol L ^-1^	5.5 x 10 ^-6^ mol L ^-1^	80	[35]
Human serum	Differential pulse polarography	9.5 x 10 ^-6^ -1.2 x 10 ^-4^ mol L ^-1^	5.5 x 10 ^-6^ mol L ^-1^	85	[35]
Pharmaceuticals	Spectrophotometry	2.48-34.78 μg mL ^-1^	2.5 μg mL ^-1^	-	[36]
Blood-serum	HPLC	0.1-20 μg mL ^-1^	0.05 μg mL ^-1^	88-103	[37
Animal drinking water	HPLC	10-1000 μg L ^-1^	3.5 μg L ^-1^	86.7-112.6	[38]
Food samples	Modified microelectrode	0.5-50 μM	87 nM	97.8-105.1	[39]
Swine wastewater	Fluorescence Spectrophotometry	0-1.478 μg mL ^-1^	0.149 μg mL ^-1^	102.32-120.92	[40]
Milk	Spectrofluorimetry with SiQD	0.92-9.2 μg mL ^-1^ (2-20 μM)	0.19 μg mL ^-1^ (0.43 μM)	98.8-100.5	This study

## 4. Conclusion

In this research, the water-soluble fluorescent SiQDs were synthesized based on the reaction of 3-Aminopropyltriethoxysilane and sodium citrate as precursors by one-pot hydrothermal process. The obtained results showed that fluorescence of SiQDs could be quenched due to the IFE mechanism between OTC and QDs. Synthesized and characterized quantum dots were successfully applied as a fluorescence IFE probe for the determination of OTC in milk samples. Satisfactory recoveries (98.8–100.5%) with low RSD% values (0.93–2.31%) were achieved.

The proposed method can be used as a promising tool for OTC analysis in food safety with its simple, selective, sensitive, rapid, and cheap features.
